# Jejunal diverticulitis as a rare cause of abdominal pain: a case report

**DOI:** 10.11604/pamj.2022.41.222.29095

**Published:** 2022-03-17

**Authors:** Amal Khsiba, Samir Bradai, Moufida Mahmoudi, Asma Ben Mohamed, Jawhar Bradai, Khaled Bouzaidi, Medhioub Mona, Lamine Hamzaoui, Mohamed Mousadek Azouz

**Affiliations:** 1 Gastroenterology Department, Mohamed Tahar Maamouri Hospital, Nabeul, Tunisia,; 2 Radiology Department, Mohamed Tahar Maamouri Hospital, Nabeul, Tunisia

**Keywords:** Abdominal pain, jejunal diverticulitis, antibiotic therapy, case report

## Abstract

Jejunal diverticulitis is an uncommon and underdiagnosed condition. Due to the rarity of This disease, diagnosis is often difficult and delayed. Medical treatment is usually sufficient for jejunal diverticulitis without peritonitis. Surgery is required in case of generalized peritonitis or voluminous abscess complicating diverticulitis. We report the case of a 76-year-old woman who suffered from recent abdominal pain. Diagnosis of uncomplicated jejunal diverticulitis was based on computed tomography (CT) scan. The evolution was favorable after antibiotic treatment. Jejunal diverticulitis have to be evoked among the differential diagnosis of patients with abdominal pain especially in the elderly and it is important for clinicians and radiologists to have awareness about this disease.

## Introduction

Although diverticula of the gastrointestinal tract are common in the colon, they are relatively rare in the small intestine and predominantly involve the proximal jejunum [1]. The course is usually asymptomatic, but complications are not exceptional. Acute diverticulitis is the most common presentation, but other complications can occur, mainly perforation, bowel obstruction and hemorrhage [2,3]. Diagnosis is often difficult and delayed due to its relative rarity and non-specific clinical symptoms which increase the mortality and morbidity of this pathology. We report a case of jejunal diverticulitis and we performed a literature review regarding this rare condition.

## Patient and observation

**Patient information:** a 76-year-old woman with a history of high blood pressure presented to the hospital with abdominal pain that predominates in the left upper quadrant and vomiting. The pain was moderate without irradiation. The transit was normal.

**Clinical findings:** her vital signs were normal with a temperature of 37.8°C, a heart rate of 65 beats per minute, blood pressure at 130/70 mm Hg, and saturation 98% on room air. Abdominal examination found a tenderness of the left hypochondrium with no distension. There were no palpable masses or organomegaly.

**Timeline of the current episode:** this was the first episode, and have never presented such clinical manifestations. The episode began ten days before her admission.

**Figure 1:**
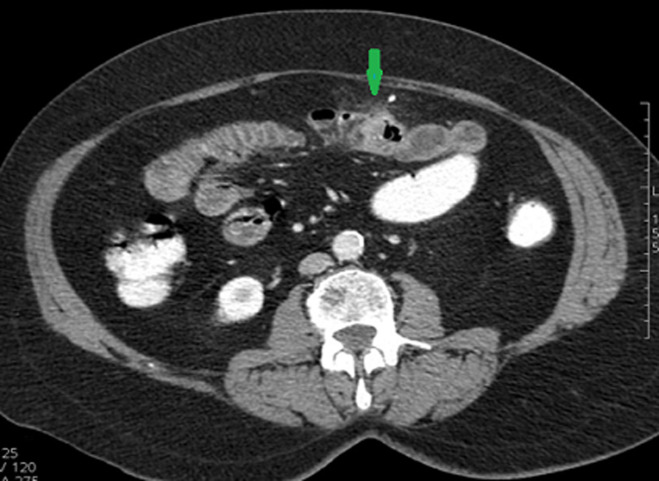
CT-scan (axial view) showing two large jejunal diverticula with wall thickening of the affected segment and the infiltration of the surrounding mesenteric fat

**Figure 2:**
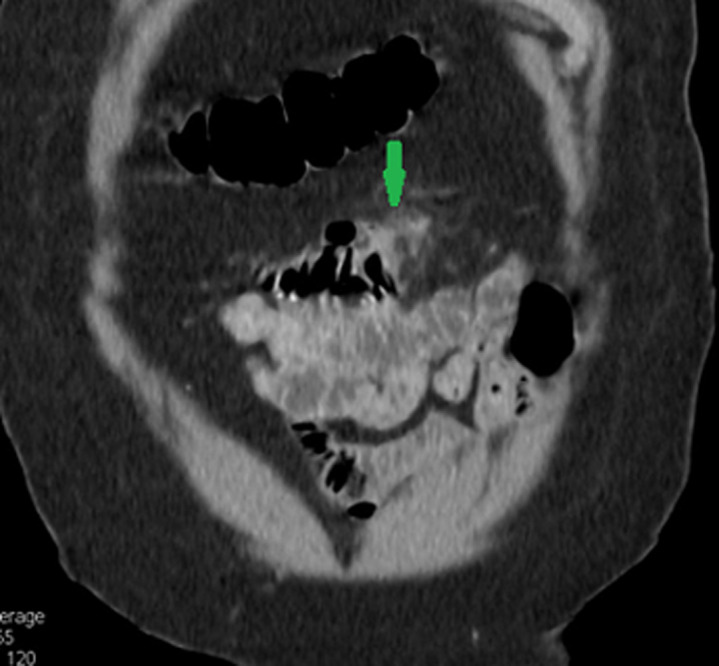
CT-scan (coronal view) showing a surrounding significant inflammatory reaction

**Diagnostic assessment:** the white blood cell count, pancreatic enzymes and liver function tests were in the normal range. C-reactive protein was 14 mg/L (range 0-5 mg/l). Patient underwent oeso-gastro-duodenal endosopy and colonoscopy which were normal. We performed an abdominal ultrasound which revealed wall thickening of a small bowel segment in the mid left abdomen. We completed with abdominal computed tomography (CT) scan which showed two large diverticula arising from the mesenteric border of jejunum with wall thickening of the affected segment with a surrounding significant inflammatory reaction and the infiltration of the surrounding mesenteric fat. No abscess formation or fluid collection was seen (Figure 1, Figure 2).

**Diagnosis:** the diagnosis of uncomplicated Jejunal diverticulitis was retained.

**Therapeutic interventions:** the patient was hospitalized and received intravenous antibiotic therapy with ceftriaxone and metronidazole during 10 days in addition to analgesics.

**Follow-up and outcome of interventions:** the course was marked by the disappearance of pain and vomiting. The patient has been followed up regularly and she is currently doing well, two years after the episode without any complication. No recurrence has been diagnosed.

**Patient perspective:** she was satisfied with the diagnostic and the proposed care.

**Patient consent:** she has given her consent for her images and other clinical informations to be reported in the journal. The patient understands that her name and initials will not be published.

## Discussion

Acquired diverticula may be primary or secondary essentially to abdominal surgery, tuberculosis, scleroderma, Crohn´s disease or other conditions. Primary acquired small bowel diverticulum, as in our case, is defined as the herniation of mucosa and submucosa through the muscular layer of the bowel at the vasa recta penetration sites [3]. They probably result from motor dysfunction of the smooth muscle or the myenteric plexus in the small bowel with abnormalities in peristalsis and disordered contractions of the affected small bowel which induce high segmental intraluminal pressures favoring la formation of diverticula [3].

Jejunal diverticular disease is a rare condition with an incidence that varies between 0.3% and 2.3% [4]. Jejunal diverticula are usually multiple [5]. Associated colonic diverticulosis is a common condition reaching up to 80% of cases [6]. However, our case was not associated with colonic diverticulosis. Jejunal diverticula occur most often in the elderly, as in our case, and they are rare before the age of 40 years [4,7]. The sex ratio is variable because of the low incidence of the disease and the low number of cases in series.

Most patients with jejunal diverticular are asymptomatic or present non specific gastrointestinal symptoms with a chance discovery during autopsy, laparotomy or imagery [2,3]. Complications can reveal the disease in 10%-20% of cases [3]. The jejunal diverticulitis is the most frequent complication with an incidence of 2% to 6% [1], but diverticulitis is much less common in the jejunum than in colonic diverticula probably because of diverticulum larger size, better intra-luminal flow and relatively sterile jejunal content [8]. Other complications are represented by perforation, bowel obstruction, pseudo obstruction, diverticular bleeding and malabsorption syndrome associated with diarrhea related to bacterial overgrowth [2,5]. Given its rarity and specific symptoms, jejunal diverticulitis is easily misdiagnosed as appendicitis, cholecystitis, peptic ulcer, Crohn’s disease or colonic diverticulitis. Therefore, clinicians must be aware of this entity to avoid misdiagnosis and treatment delay.

Ultrasound is usually used as the first-line investigation tool for abdominal pain due to its low cost, it can inconstantly reveal thickening of the intestinal wall, hypoechoic irregular formations with hyperechoic centre connected to the intestine evocative of diverticula, hyperechoic tissue around the diverticula indicating infiltration of the surrounding fat or collections with air bubbles [1].

Computed tomography (CT) scan of the abdomen plays a crucial role in the diagnosis of jejunal diverticulitis as well as its complications. The CT scan of jejunal diverticulitis typically shows an inflammatory process, wall thickening of the affected segment, infiltration of the surrounding mesenteric fat or an abscess adjacent to a jejunal loop [1,2]. The inflammation can affect concomitantly more than one diverticulum and it can be rarely extensive [8]. Patients with jejunal diverticulosis can present a pneumoperitoneum without any peritonitis as the diverticulum thin wall may behave as a semipermeable membrane which allows the passage of air [8]. The diagnosis remains uncertain, especially in the severe or locally advanced forms, in which the identification of the pathological diverticulum can be difficult because of the importance of local liquid and/or gas infiltration. But, the predominance of abnormalities on the mesenteric edge of the loop small bowel and the association with other jejuno-ileal diverticula suggest the diagnostic [9].

Therapeutic management of acute jejunal diverticulitis is not consensual and it is based on the experience of the different teams [4]. The treatment for uncomplicated jejunal diverticulitis should be conservative combining intravenous antibiotics and fasting [4,7,8]. Similarly, for localized contained perforation, a conservative management may be indicated in hemodynamically stable patients [4,7]. If there is no clinical improvement within 48-72h, surgery should be discussed. In cases of peri-diverticular abscess, a non-surgical treatment combining intravenous antibiotics and CT-guided drainage depending on the size of the collection and feasibility of percutaneous drainage (when necessary) may be sufficient [7,8]. Surgical resection of the affected digestive segment with primary anastomosis is the reference treatment of complicated diverticulum (haemorrhage, obstruction, perforation) [4,7]. In case of extensive jejunal diverticulosis, resection should be limited to the the affected diverticulum to avoid short bowel syndrome, in addition, the risk of occurrence in other sites is unknown [7,10]. Asymptomatic patients with diverticula fortuitously discovered by imaging or during surgery require no specific intervention [8].

## Conclusion

Jejunal diverticulitis is an uncommun cause of abdominal pain especially in the elderly. It is often overlooked by the clinician. The diagnosis must be early because the perforation is associated with a high morbidity and mortality. It is therefore important to recognize and include this entity in the differential diagnosis of patients with abdominal pain. Computed tomography imaging aids in diagnosis, excludes other causes of abdominal pain, assesses the extent of the disease and its complications. The therapeutic option depends on the terrain, the severity of the clinical and radiological signs and the evolution.
